# Myocardial deformation in malignant mitral valve prolapse: A shifting paradigm to dynamic mitral valve–ventricular interactions

**DOI:** 10.3389/fcvm.2023.1140216

**Published:** 2023-04-12

**Authors:** Nathalie Pace, Jean-Marc Sellal, Clement Venner, Damien Mandry, Pierre-Yves Marie, Laura Filippetti, Mathieu Echivard, Antoine Fraix, Nicolas Girerd, Zohra Lamiral, Christian De Chillou, Nicolas Sadoul, Christine Selton-Suty, Olivier Huttin

**Affiliations:** ^1^ Department of Cardiology, Nancy University Hospital, Vandoeuvre les Nancy, France; ^2^ Cardiology Department, Clinique Saint Augustin, Bordeaux, France; ^3^ Department of Nuclear Medicine, Nancy University Hospital, Vandoeuvre les Nancy, France; ^4^ Nuclear Medicine Department, Université de Lorraine, INSERM, Nancy, France; ^5^ Department of Radiology, CHRU-Nancy, Nancy, France; ^6^ IADI, INSERM, Université de Lorraine, Nancy, France; ^7^ INSERM Centre d’Investigation Clinique CIC-P 9501, Nancy University Hospital, Vandoeuvre les Nancy, France

**Keywords:** mitral valve prolapse (MVP), sudden cardiac death (SCD), ventricular arrhythmia (VA), speckle tracking echocardiography, myocardial deformation imaging

## Abstract

**Objectives:**

This study sought to assess the value of myocardial deformation using strain echocardiography in patients with mitral valve prolapse (MVP) and severe ventricular arrhythmia and to evaluate its impact on rhythmic risk stratification.

**Background:**

MVP is a common valvular affection with an overly benign course. Unpredictably, selected patients will present severe ventricular arrhythmia.

**Methods:**

Patients with MVP as the only cause of aborted SCD (MVP-aSCD: ventricular fibrillation and monomorphic and polymorphic ventricular tachycardia) with no other obvious reversible cause were identified. Nonconsecutive patients referred for the echocardiographic evaluation of MVP were enrolled as a control cohort and dichotomized according to the presence or absence of premature ventricular contractions (MVP-PVC or MVP-No PVC, respectively). All patients had a comprehensive strain assessment of mechanical dispersion (MD), postsystolic shortening, and postsystolic index (PSI).

**Results:**

A total of 260 patients were enrolled (20 MVP-aSCD, 54 MVP-PVC, and 186 MVP-No PVC). Deformation pattern discrepancies were observed with a higher PSI value in MVP-aSCD than that in MVP-PVC (4.6 ± 2.0 vs. 2.9 ± 3.7, *p* = 0.014) and a higher MD value than that in MVP-No PVC (46.0 ± 13.0 vs. 36.4 ± 10.8, *p* = 0.002). In addition, PSI and MD increased the prediction of severe ventricular arrhythmia on top of classical risk factors in MVP. Net reclassification improvement was 61% (*p* = 0.008) for PSI and 71% (*p* = 0.001) for MD.

**Conclusions:**

In MVP, myocardial deformation analysis with strain echocardiography identified specific contraction patterns with postsystolic shortening leading to increased values of PSI and MD, translating the importance of mitral valve–myocardial interactions in the arrhythmogenesis of severe ventricular arrhythmia. Strain echocardiography may provide important implications for rhythmic risk stratification in MVP.

## Introduction

Mitral valve prolapse (MVP) is a common echocardiographic finding (prevalence of approximately 2%) that has an overall benign course (1, 2). However, selected patients will present ventricular arrhythmia or even sudden cardiac death (SCD), for which the underlying mechanism remains unpredictable (1–4).

Several MVP phenotypes have been described and correlated to an increased risk of arrhythmias: bileaflet prolapse, female sex, ectopic ventricular activity, and ST-segment/T-wave anomalies (5–8). These risk factors lack specificity in discerning a clinically relevant subset of higher-risk patients.

More recently, cardiac magnetic resonance (CMR) imaging has provided a new focus on fibrosis induced by recurring the stretch exerted by the prolapsing leaflet on the myocardium (9, 10). Moreover, mitral annular anomalies such as disjunction (MAD) or curling provided a dynamic substrate that might aggravate the fibrotic process and facilitate the occurrence of ventricular arrhythmia (11–16).

New insights were provided in assessing the interactions between the mitral valve and the left ventricle (LV) using speckle-tracking strain echocardiography in MVP (17). Regional disparities of myocardial deformation with abnormal postsystolic shortenings have been identified in the mitral annular regions and the surroundings of the insertion sites of the papillary muscles (18). These regions may represent a potential trigger for ventricular arrhythmia.

The aims of our study were to assess myocardial deformation using strain echocardiography in patients with MVP and a history of aborted SCD, to compare this assessment with patients with MVP presenting with and without premature ventricular contraction, and to evaluate the value of strain echocardiography on top of established risk factors for arrhythmic risk stratification.

## Materials and methods

### Selection of MVP patients with aborted SCD

All patients who had an indication for an internal cardioverter defibrillator (ICD) between 2000 and 2018 were retrospectively selected. Patients with a prophylactic indication were discarded. Secondary indications were guideline-directed and consisted of aborted sudden cardiac death (SCD: ventricular fibrillation and monomorphic and polymorphic ventricular tachycardia) or syncope aggravating a documented cardiomyopathy. Patients underwent a comprehensive cardiac evaluation with systematic echocardiography, coronary angiography, cardiac MRI, and electrophysiological study when judged necessary.

**Figure 1:**
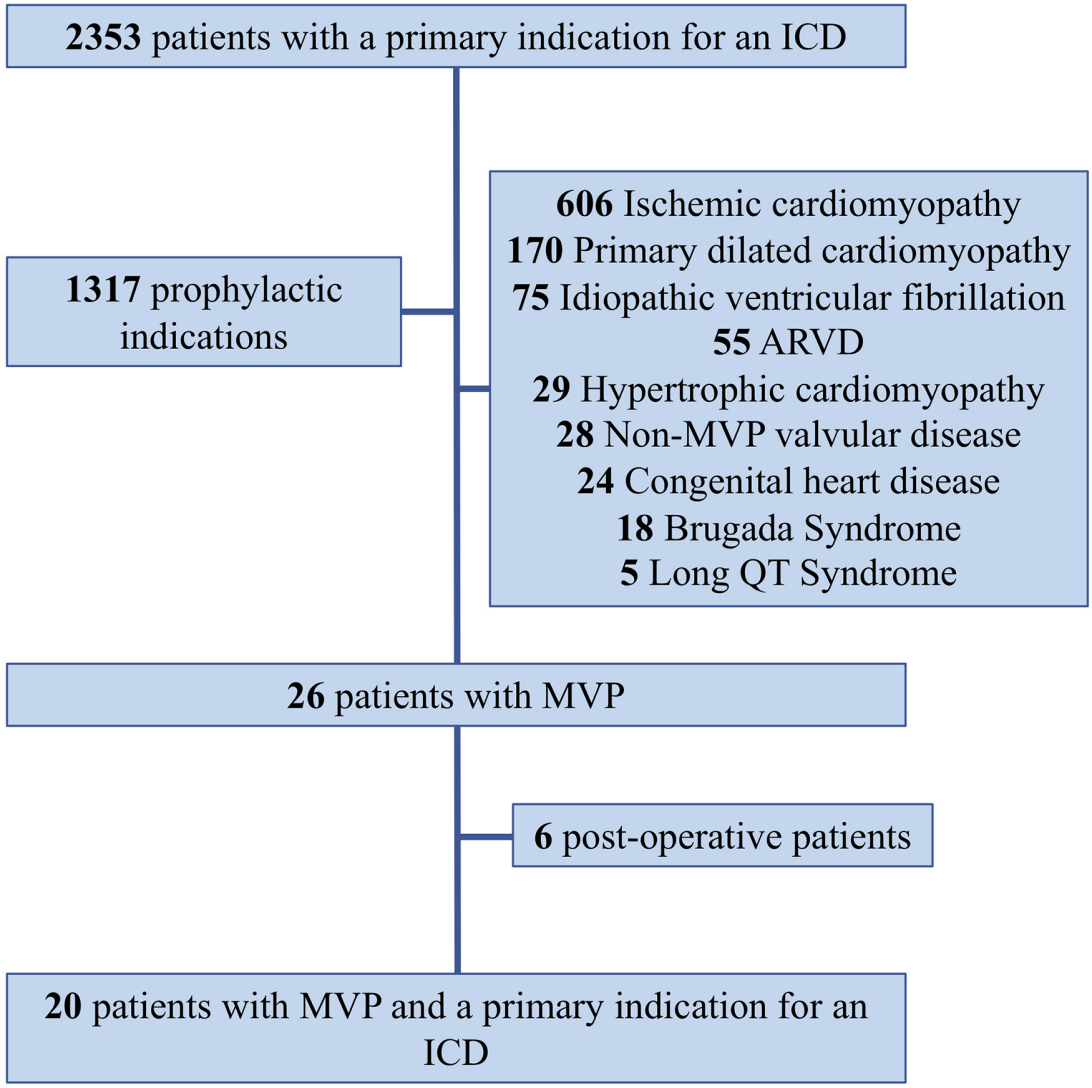
Patient selection flowchart. ARVD, arrhythmogenic right ventricular dysplasia; ICD, internal cardioverter defibrillator.

Mitral valve prolapse was implicated when a reversible cause was carefully excluded after the patient's file review and in the absence of any of the following: any obstructive coronary artery disease, structural cardiomyopathy or impaired LV function (ejection fraction <50%), active myocarditis, long QT syndrome, or channelopathy. For the purpose of the study, patients who underwent mitral valve surgery were not included in the analysis (Figure 1).

To account for potential residual postresuscitation myocardial electromechanical discrepancies, only echocardiography during patients' follow-ups was analyzed.

### Selection of control MVP patients

A nonconsecutive control cohort of patients with MVP was retrospectively selected from our echocardiography database. Only comprehensive echocardiography that allowed *ad hoc* postprocessing speckle-tracking analysis and mitral valve characterization was left for analysis. The clinical investigation focused on MVP-related symptoms (chest pain, palpitations, lipothymia, or syncope) and functional status. The presence of premature ventricular contraction (PVC) was based on 24-h external loop recorders.

The investigation was in line with the Declaration of Helsinki and good clinical practice guidelines.

### Echocardiography and speckle-tracking evaluation

Echocardiography was performed using a commercially available standard ultrasound scanner (Vivid 9 and E95; General Electrics Vingmed Ultrasound, Horten, Norway) with a 2.5-MHz transducer.

MVP was defined as a systolic displacement of the mitral leaflet >2 mm beyond the annular plane, and mitral regurgitation (MR) was graded according to recent guidelines (19). The location and etiology of MVP were evaluated from the parasternal and apical views.

Mitral annular disjunction (MAD) was measured in the parasternal long axis as the maximal distance between the insertion of the mitral valve leaflet and the inferolateral basal LV wall. Curling of the basal myocardium, which represents the systolic rocking motion of the inferolateral basal LV wall, was measured as the length of a perpendicular line joining the tip of the inferolateral wall to the insertion of the mitral valve leaflet, as previously described (15, 16). LV ejection fraction (LVEF) was measured for each patient by Simpson's biplane method. LV segmentation was defined according to a 17-segment ACC/AHA model.

Speckle-tracking analysis was performed offline by investigators blinded to the arrhythmic status of the patients using automated and dedicated software (Q analysis software, Echo PAC PC version 110.1.0, GE Healthcare) from the three apical views. Myocardial longitudinal deformation curves were obtained with measurement for each segment of (1) maximal absolute value of strain during the ejection phase before aortic valve closure (peak global longitudinal strain: GLS) and (2) postsystolic strain (PSS) as the maximal absolute value of strain during isovolumic relaxation after the aortic valve closure with the calculation of the PSS index (PSI) (18). Values were obtained for each of the 17 segments and averaged to obtain global values. Contraction duration was calculated as the time from the ECG onset of the Q or R waves to the peak negative strain for each of the 17 segments. Mechanical dispersion (MD) was defined as the standard deviation of the contraction durations. Bull's eyes representations with automatic display of GLS and PSI values were also obtained for visual representation.

### Statistical analysis

Normally distributed values were expressed as mean ± SD and compared using Student's *t*-tests. Data not normally distributed were presented as median (25th–75th or interquartile range) and compared using Mann–Whitney *U* tests. Categorical variables were expressed as a percentage and were compared using Pearson's chi-squared tests.

Univariable logistic regression was performed to assess the association between aborted SCD as an outcome and each of the explanatory variables: age, female sex, PVC, chest pain, lipothymia/syncope, familial SCD, bileaflet prolapse, Barlow's disease, LVEF, LV end-systolic diameter, left atrial end-systolic volume, curling, MAD, MR >2, GLS, PSI, and mechanical dispersion. Odds ratios and their confidence intervals were reported.

Multivariable logistic regression models were used to estimate the association between aborted SCD and each of the deformation parameters (GLS, PSI, and MD) after adjustment for the previous and significant explanatory variables from the univariable analysis. Odds ratios and their confidence intervals were reported.

The incremental value of the adjunction of deformation parameters on top of classical risk factors (age, female sex, PVC, bileaflet prolapse*,* MR severity, and LVEF) was assessed using net reclassification improvement (NRI). For this purpose, GLS was considered as a continuous variable, PSI as a binary variable (dichotomized according to the median value of distribution in the global MVP population: PSI >4), and MD in a logarithmic scale. SAS version 9.4 was used for the statistical analysis. *P* values ≤ 0.05 were considered statistically significant.

## Results

### Study population characteristics

Twenty patients with MVP had an indication for secondary ICD based on the occurrence of an aborted SCD (aSCD), or equivalent, yielding a 2% incidence. Age ranged from 15.1 to 71.1 years. There were 11 female patients (55%). All data concerning MVP-aSCD patients are presented in Table 1 and Supplementary Table S1.

**Table 1:** Clinical and echocardiographic characteristics of the global population and comparison according to rhythmic profile.

	MVP patients				Global *p* values
	Overall *N* = 240	MVP-No PVC *N* = 186	MVP-PVC *N* = 54	MVP-aSCD *N* = 20	
**Clinical characteristics**					
Age	57.4 ± 16.5	57.3 ± 17	57.9 ± 14.8	48.1 ± 18.5	0.056
Male sex	144 (60)	115 (61.8)	29 (53.7)	9 (45)	0.240
**Symptoms**					
Chest pain	35 (16.1)	20 (11.7)	15 (32.6)	1 (5)	0.001
Pre/syncope	10 (4.9)	4 (2.4)	6 (14.6)	8 (40)	<0.001
NYHA					0.014
I/II	173 (72.4)	131 (70.4)	43 (79.7)	20 (100)	
III/IV	66 (27.6)	55 (29.6)	11 (20.4)	0 (0)	
**Atrial fibrillation**					
Paroxysmal	28 (11.7)	23 (12.4)	5 (9.3)	2 (10)	0.800
Permanent	11 (4.6)	10 (5.4)	1 (1.9)	1 (5)	0.552
**Treatment**					
Beta-blocker	63 (26.8)	44 (23.7)	19 (35.2)	14 (70)	<0.001
Amiodarone	19 (8.1)	18 (9.7)	1 (1.9)	1 (5)	0.196
**MVP characterization**					
Etiology					0.010
FED	149 (90.3)	52 (28.0)	48 (88.9)	16 (80)	
Barlow	16 (9.7)	16 (8.6)	6 (11.1)	4 (20)	
Mitral regurgitation					0.005
≤2	86 (35.9)	65 (35)	21 (38.9)	15 (75)	
3–4	154 (64.2)	121 (65)	33 (61.1)	5 (25)	
**Prolapsing leaflet**					
Posterior	214 (89.2)	166 (89.2)	48 (88.9)	15 (75)	0.480
Anterior	117 (48.8)	89 (47.8)	28 (51.9)	16 (80)	0.526
Bileaflet	90 (37.5)	68 (36.6)	22 (40.7)	11 (55)	0.261
Flail leaflet	112 (46.7)	94 (50.5)	18 (33.3)	2 (10)	0.001
Annular disjunction	93 (38.8)	73 (39.2)	20 (37)	9 (45)	0.824
MAD (mm)	8.5 ± 3.8	8.2 ± 3.9	9.7 ± 2.9	9.9 ± 4.0	0.167
Curling (mm)	6.0 ± 2.3	5.7 ± 2.4	7.1 ± 1.8	9.2 ± 4.7^#^	<0.001
**Ventricular function**					
LVEDD	55.4 ± 7.6	55.1 ± 7.7	56.3 ± 7.5	52.6 ± 7.1	0.191
LVEDV	141.0 ± 45.0	138.2 ± 45.5	150.5 ± 42.0	122.4 ± 35.5^†^	0.040
LVESD	35.0 ± 6.1	34.7 ± 6.0	35.4 ± 6.2	35.8 ± 5.0	0.604
LVESV	48.3 ± 18.9	47.1 ± 18.8	52.7 ± 18.7	50.9 ± 14.7	0.125
EF	65.6 ± 7.3	65.8 ± 7.3	64.8 ± 7.6	58.4 ± 4.7^#†^	<0.001
LAESD	60.7 ± 32.0	60.7 ± 33	60.7 ± 28.4	48.5 ± 33.9	0.292
TAPSE	25.6 ± 5.3	25.4 ± 5.5	26.3 ± 4.2	25.6 ± 4.3	0.555
sPAP	30.5 ± 13.3	30.9 ± 13.7	28.9 ± 11.8	22.8 ± 7.7	0.066
**Strain echocardiography**					
GLS (%)	−21.3 ± 3.3	−21.4 ± 3.3	−21.2 ± 3.5	−18.6 ± 3.1^#†^	0.003
PSS (%)	−21.5 ± 3.2	−21.6 ± 3.2	−21.4 ± 3.5	−19.0 ± 3.2^#†^	0.005
PSI	3.3 ± 2.9	3.5 ± 2.6	2.9 ± 3.7	4.6 ± 2.0^†^	0.014
MD (ms)	37.4 ± 12.9	36.4 ± 10.8	40.9 ± 18.2	46.0 ± 13.0^#^	0.002

Values are expressed as mean ± SD or *n* (%).

*p < 0.05 MVP-PVC vs. MVP-No PVC.

†*p* < 0.05 MVP-aSCD vs. MVP-PVC.

^#^ *p* < 0.05 MVP-aSCD vs. MVP-No PVC.

FED, fibroelastic deficiency; GLS, global longitudinal strain; LAESV, left atrial end-systolic volume (ml/m^2^); LVEDD, LV end-diastolic diameter (mm); LVEDV, LV end-diastolic volume (ml); LVEF, LV ejection fraction; LVESD, LV end-systolic diameter (mm); LVESV, LV end-systolic volume (ml); NYHA, New York Heart Association; MAD, mitral annular disjunction; MD, mechanical dispersion; PSI, postsystolic strain index; PSS, postsystolic strain; PVC, premature ventricular contraction; sPAP, systolic pulmonary artery pressure (mmHg).

aSCD was the inaugural event leading to the diagnosis of MVP for eight (40%) patients. MVP corresponded to an incidence of 2% of secondary indications for overall ICD implantation. Indications were based on monomorphic ventricular tachycardia (6 patients, 30%), polymorphic tachycardia (1 patient, 5%), and ventricular fibrillation (13 patients, 65%).

Fifty-five percent of patients had no mitral annular disjunction. CMR was performed in 15 patients (75%). Eleven (73%) patients had LV myocardial fibrosis, and eight (53%) patients had papillary muscle fibrosis.

Following the aSCD event, an ICD was systematically suggested. Seventeen patients (85%) accepted the implantation. At least one recurrent arrhythmic event requiring appropriate ICD therapy occurred in 9out of 17 implanted patients (53%) after a mean delay of 1.6 ± 2.2 years. Seventy-eight percent of recurrence occurred within the first year after ICD implantation. No patient died during a mean follow-up of 5.1 years. Two patients (10%) underwent mitral valve surgery for symptomatic MR.

The control MVP population consisted of 240 patients with no difference in regard to age and sex. The control population was dichotomized on the presence (MVP-PVC) or absence (MVP-No PVC) of PVC. PVC was documented in 54 patients (22.5%). Clinical parameters are presented in Table 1.

### Comparison according to rhythmic presentation

Comparative clinical and echocardiographic data are listed in Table 1. MVP-aSCD patients exhibited a higher rate of atypical symptoms (chest pain, *p* = 0.001; presyncope or syncope, *p* < 0.001), while others had more frequent MR-related symptoms (dyspnoea, *p* = 0.014).

**Figure 2:**
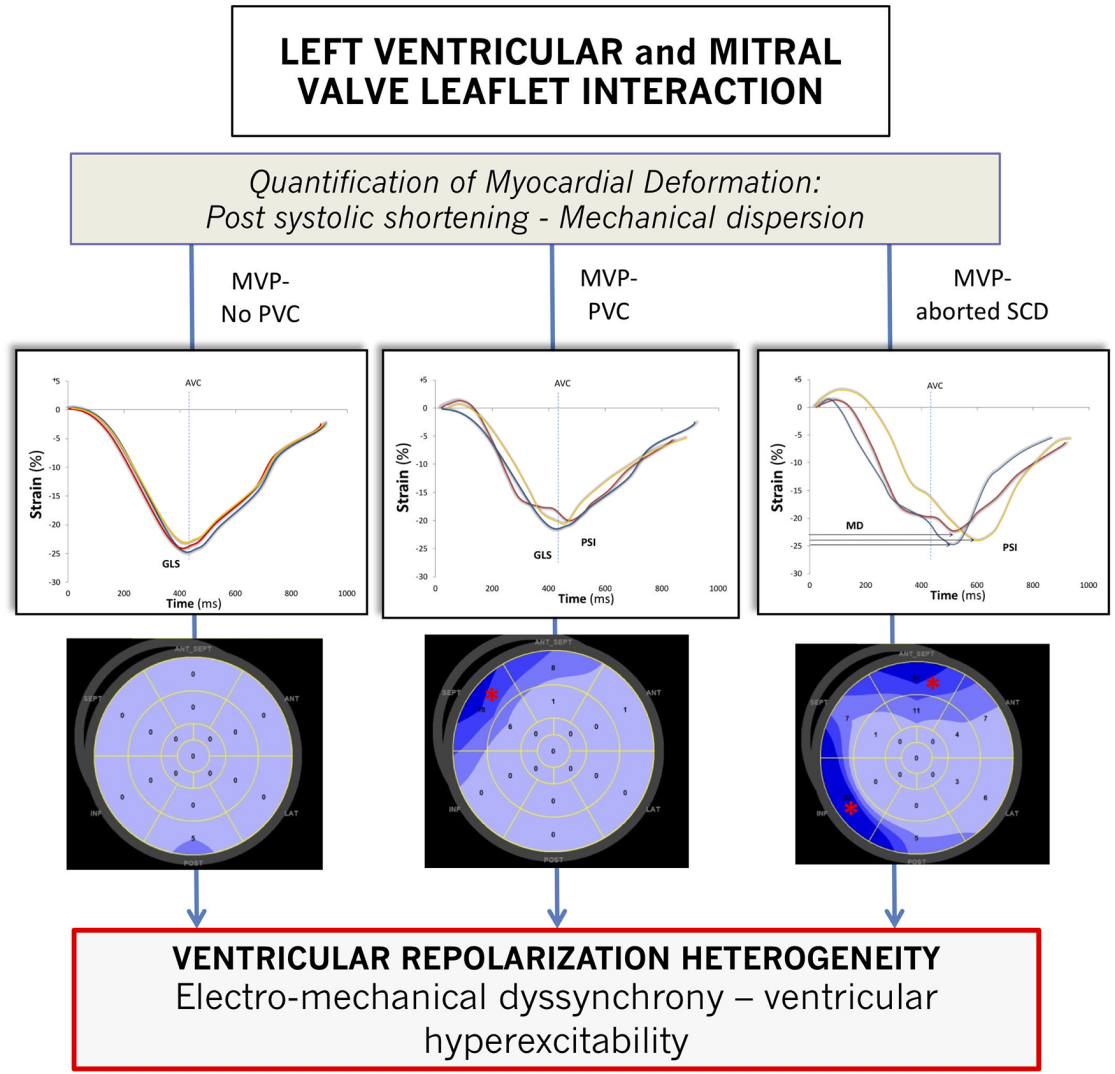
Strain curve profiles according to the importance of prolapse-induced exaggeration of myocardial deformation along with a global representation (bull's eye view) of myocardial segments with postsystolic shortening. AVC, aortic valve closure; GLS, global longitudinal strain; MD, mechanical dispersion. * indicates segments with postsystolic shortening. For a visual representation, only three deformation curves are displayed: basal anteroseptal segment (red curve), mid-anteroseptal segment (blue curve), and basal inferolateral segment (yellow curve).

Higher-grade MR (3, 4) was predominant among MVP-No PVC patients (*p* = 0.005) with more frequent flail leaflet (*p* = 0.001), increased LV end-diastolic volume (*p* = 0.040), EF (*p* < 0.001), and global longitudinal strain (*p* = 0.003). There was no difference in the presence of mitral annular disjunction among the different groups of patients (*p* = 0.824). However, curling of the inferolateral basal wall increased significantly (*p* = 0.001) in MVP-aSCD vs. MVP-PVC and MVP-PVC vs. MVP-No. A linear and significant correlation was observed between MAD and curling (*r* = 0.87, *p* < 0.001). Significant differences in PSS (*p* = 0.005) and MD (*p* = 0.002) were noted, with higher values observed among MVP-aSCD vs. MVP-No PVC (Figure 2).

### Prognostic assessment in regard to the risk of SCD in MVP patients

Associations between aborted SCD and classical risk factors of ventricular arrhythmia in MVP were studied in a univariate analysis (Table 2). Multiple variables correlated to the occurrence of SCD, including documented PVC (OR 3.36 [1.35–8.35], *p* = 0.009) or pre/syncope (OR 9.8 [3.24–29.67], *p* < 0.001), curling (OR 1.41 [1.07–1.87], *p* = 0.016), drop in GLS values (OR 1.26 [1.10–1.45], *p* = 0.001), and higher MD (OR 1.04 [1.01–1.06], *p* = 0.013). Additional values of GLS, PSI, and MD were independently assessed in multivariable models after adjustment for pairs of established risk factors (age, PVC, female sex, MR grade, Barlow's phenotype, pre/syncope, presence of MAD, and curling) (Table 3).

**Table 2:** Univariable analysis of clinical and echocardiographic parameters for the risk of SCD.

	Univariable		
Variable	OR	IC 95%	*P* values
Age	0.97	0.94–0.99	0.014
Female sex	2	0.81–4.93	0.132
PVC	3.36	1.35–8.35	0.009
Pre/syncope	9.80	3.24–29.67	<0.001
Familial SCD	3.56	0.67–18.88	0.136
Bileaflet prolapse	2.22	0.91–5.48	0.083
Barlow	2.40	0.74–7.80	0.146
LVEF	0.87	0.81–0.93	<0.001
LVESD	1	0.70–1.41	0.977
MAD	1.29	0.52–3.24	0.583
Curling	1.41	1.07–1.87	0.016
MR >2	0.22	0.08–0.60	0.003
GLS	1.26	1.10–1.45	0.001
PSI	1.15	1.01–1.32	0.043
MD	1.04	1.01–1.06	0.013

Curling and MAD were considered categorical variables. GLS, PSI, and MD were considered continuous variables.

GLS, global longitudinal strain; LVEF, LV ejection fraction; LVESD, LV end-systolic diameter; LAESV, Left atrial end-systolic volume; MAD, mitral annular disjunction; MD, mechanical dispersion; MR, mitral regurgitation; PSI, postsystolic strain index; PVC, premature ventricular contraction.

**Table 3:** Multivariate analysis of deformation parameters (GLS, PSI, and MD) adjusted for established risk factors of SCD in MVP.

		GLS			PSI			MD		
		OR	IC 95%	*P* Values	OR	IC 95%	*P* Values	OR	IC 95%	*P* Values
**Univariable**										
	SCD	1.26	1.1–1.45	0.001	1.15	1.01–1.32	0.043	1.04	1.01–1.06	0.013
**Multivariable**										
	Adjustment variables									
Model 1	Age/LVEF	1.14	0.96–1.35	0.144	1.15	0.98–1.34	0.079	1.04	1.01–1.07	0.007
Model 2	PVC/curling	1.27	1.10–1.48	0.001	1.17	1.01–1.34	0.033	1.03	0.99–1.05	0.076
Model 3	PVC/MAD	1.27	1.09–1.48	0.001	1.17	1.01–1.34	0.033	1.03	0.96–1.03	0.774
Model 4	PVC / presyncope	1.30	1.12–1.52	0.001	1.18	1.02–1.37	0.024	1.04	1–1.07	0.031
Model 5	PVC/Barlow	1.30	1.12–1.51	0.001	1.17	1.02–1.35	0.029	1.03	1–1.06	0.046
Model 6	PVC/female sex	1.29	1.11–1.49	0.001	1.16	1.01–1.33	0.04	1.03	1–1.06	0.039
Model 7	PVC/MR >2	1.26	1.08–1.47	0.004	1.24	1.06–1.45	0.006	1.03	1–1.06	0.03

GLS, global longitudinal strain; LVEF, LV ejection fraction; MAD, mitral annular disjunction; MD, mechanical dispersion; MR, mitral regurgitation; PSI, postsystolic strain index; PVC, premature ventricular contraction.

### Improvement in risk reclassification associated with deformation parameters

**Figure 3:**
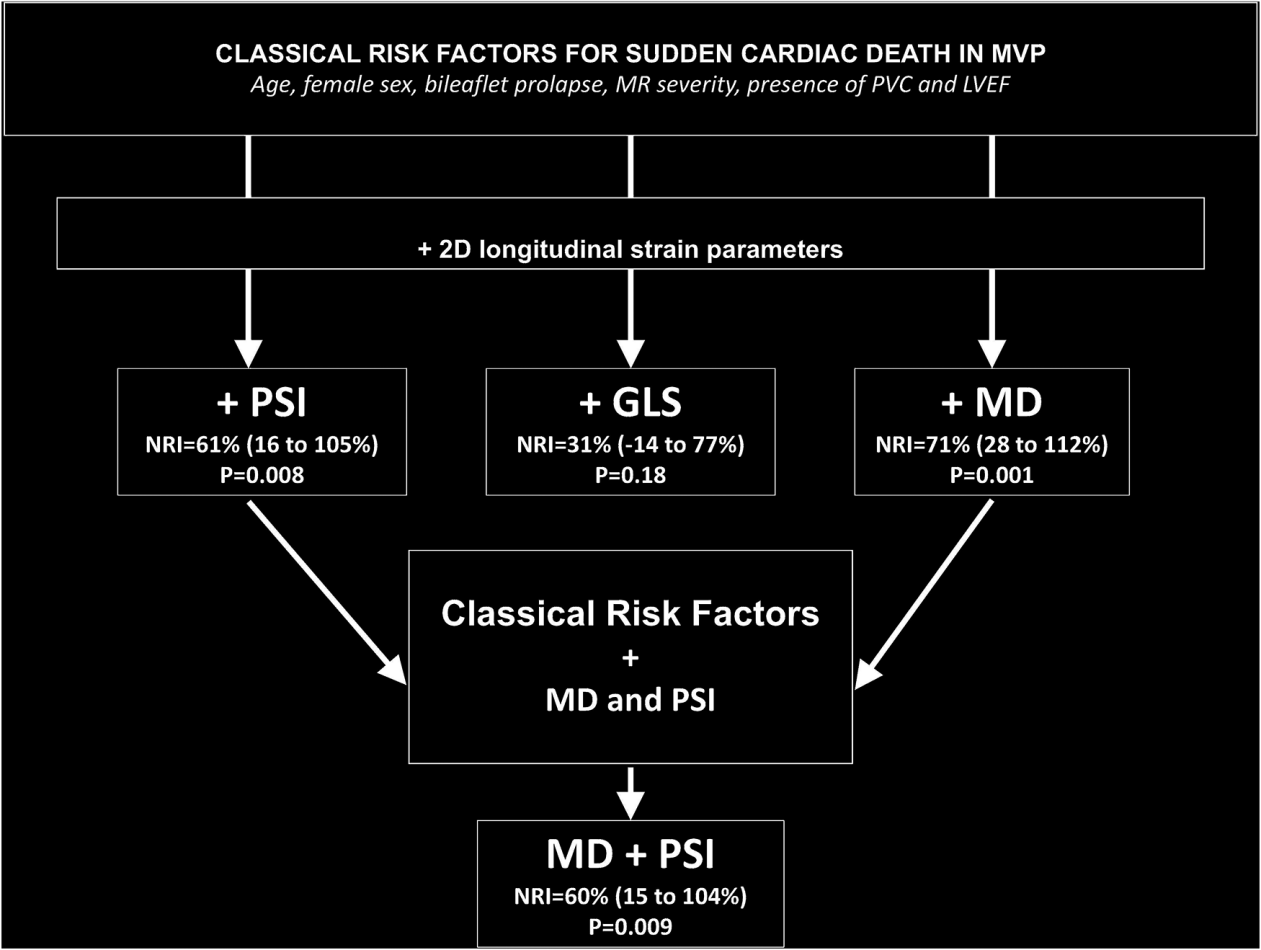
Improvement in risk reclassification associated with deformation parameters over classical risk factors in MVP. Classical risk factors include clinical features (age and female sex), valve structure (bileaflet prolapse and MR severity), the presence of PVC, and LVEF.

The increased discriminative values associated with adding GLS, PSI, and MD on top of the classical established risk factors (age, female sex, bileaflet prolapse, MR >2, PVC, and LVEF) were evaluated to predict SCD using NRI. The addition to the logistic model of PSI (NRI = 61%, *p* = 0.008) or MD (NRI = 71%, *p* = 0.001) was associated with a significant improvement of reclassification but not of GLS (NRI = 31%, *p* = 0.18). In a model containing the classical risk factors and MD, adding PSI provided further reclassification possibility (NRI = 60%, *p* = 0.009) (Figure 3; Supplementary Table S2).

## Discussion

Based upon the comparative evaluation of 20 patients with MVP who had presented an aborted SCD to a control cohort of MVP patients, our study shows (1) a 2% prevalence of MVP among secondary indications of ICD, (2) clinical and echocardiographic characteristics associated with a higher risk of severe ventricular arrhythmia, and (3) a specific strain deformation profile combining contraction inhomogeneity with postsystolic shortening and increased value of mechanical dispersion.

Strain echocardiography conveys risk reclassification information of relative importance in the arrhythmic evaluation of MVP patients on top of established risk factors. These parameters, relaying the implication of myocardial–leaflet interactions, may be of particular interest in the identification of high-risk MVP patients.

### Clinical need for further risk stratification of arrhythmic prolapses

Despite a much lower incidence with regard to ischemic cardiomyopathy, MVP-related cardiac arrests account for 2.5% of overall SCD and mainly affect a younger population (1, 2). This value has to be pondered in light of an estimated 1.2% prevalence of MVP in the general population and an SCD rate ranging from 0.1 to 0.4% per patient year (2, 20, 21).

The mechanisms behind ventricular arrhythmia in MVP are complex. Three sites at the origin of ventricular arrhythmias were identified: (1) papillary muscles, (2) mitral annulus, and (3) LV fascicules (5, 22). Most monomorphic ventricular tachycardia occurs in the presence of a significant anatomic structural substrate, such as myocardial fibrosis, which might exacerbate differences in myocardial excitability responsible for a re-entry mechanism. Nonetheless, most of our patients initially presented with ventricular fibrillation, and the presence of late-gadolinium enhancement was not constant. In this setting, both the genesis and maintenance of these polymorphic tachyarrhythmias remain unclear and are most likely a PVC-triggered mechanism (5, 22). Paradoxically, the prognostic implication of the presence and severity of PVC in MVP remains unestablished.

Various and heterogeneous causes of electrical disturbances may explain the difficulty in identifying a specific mechanism at the origin of cardiac arrhythmias. Understanding the underlying mechanism for better estimating the consequent rhythmic risk is critical in developing a large-scale screening strategy prior to discussing a specific therapeutic algorithm.

Established risk factors include female sex, PVC, bileaflet or valvular redundancy, mitral regurgitation severity, and ST-segment or T-wave anomalies, but all, individually or combined, are not specific enough to clearly identify high-risk patients (6–8). Indeed, most of our aborted SCD patients did not fulfill these conventional criteria. Leaflet redundancy, or Barlow's phenotype, is highly subjective and may concern up to 41% of MVP patients (1).

The implication of MR is also equivocal, and most of our patients had none to mild MR. Fibrosis in the setting of important MR may rather be associated with MR-induced ventricular remodeling, neurohumoral activation, and subsequent myocardial scar (4, 8, 23).

Mitral annular disjunction was recently described as another important dynamic substrate for arrhythmogenesis (16, 24). MAD is preponderantly observed in patients with MVP and is associated with papillary muscle fibrosis and severe arrhythmic event (16). MAD may also accentuate the level of myocardial stretch, particularly in the regions surrounding the mitral annulus, and generate a greater degree of intraventricular electromechanical dyssynchrony than the one intrinsically due to the MVP. However, MAD description and quantification are not standardized, and its prognostic value has mostly been reported in CMR (13, 15, 16). In our study, despite a strong and linear correlation between MAD and curling, only the latter was associated with the presence of PVC and severe arrhythmic events.

### Imaging the myocardial substrate and myocardial–mitral valve interactions

Renewed interest in malignant MVP has been stimulated by recent works based on identifying LV myocardial fibrosis (9, 23). Among 650 SCD patients with no other cardiac structural anomaly than MVP on autopsy, Basso et al. reported histologically documented fibrosis in 88% of patients, mostly located in the papillary muscles or adjacent regions and inferobasal LV wall (10). The authors further investigated the relationship between the PVC burden and fibrosis using CMR among MVP patients referred for PVC ablation, hence raising the possibility of fibrosis preceding the occurrence of ventricular arrhythmias and portraying a potential myocardial substrate. Identifying fibrosis implies the possibility of myocardial scarring related to repeated traction exerted on the myocardial wall by an excessive tension imposed on the papillary muscles from the prolapsing leaflet (25, 26).

Fibrosis appears to be a pivotal structural damage strongly associated with the occurrence of ventricular arrhythmia but is not systematically observed in SCD survivors. In a recent meta-analysis evaluating MVP and SCD, Nalliah et al. reported that 13% of patients had fibrosis on CMR without ventricular arrhythmia and 20% had VA without fibrosis (21). Fibrosis may generate re-entry circuits, which are an important determinant of ventricular arrhythmia, but other determinants are suspected to be of equal importance, such as endomyocardial friction, afterdepolarization ectopic contractions, and myocardial–mitral valve interactions (7, 15, 27).

Previous studies focusing on myocardial deformation in MVP have found that the mechanical interactions between the LV myocardium and the mitral valve can be achieved using strain echocardiography (17, 18). GLS, which only reflects the peak of deformation occurring at aortic valve closure, is not sufficient in this assessment and does not account for the important temporal changes occurring throughout the cardiac cycle, particularly during ventricular repolarization (28). Abnormal contraction patterns were identified in MVP with postsystolic shortening consisting of contractions occurring after aortic valve closure. MD reflects on electromechanical dyssynchrony, hence on the amount of post-systolic shortening segments, and relates to the presence of histological alteration, such as fibrosis. Its value increases along with the intensity of the heterogeneity of ventricular depolarization–repolarization and is associated with the occurrence of ventricular arrhythmias in other affections, such as ischemic cardiomyopathy or aortic stenosis (29, 30).

Deformation analysis with a specific focus on postsystolic shortening and MD bore significant supplemental prognostic information in arrhythmic risk reclassification on top of all accepted classical risk factors of SCD. Fibrosis detection using strain analysis has proven reliable, especially when using PSI (31). However, the correlation between increased values of PSI or MD and the extent of fibrosis has never been studied in the context of MVP.

### Malignant mitral valve prolapse: One or many sides to the same affection?

The connection between mitral valve prolapse and ventricular arrhythmia has evolved from the initially described ballerina-foot pattern to the identification of myocardial fibrosis in cardiac MRI and the description of MAD and the concept of myocardial stretch (10, 12, 25, 32).

As we have described, patients with aSCD showed a higher degree of myocardial dyssynchrony compared to patients with PVC and control MVP patients, suggesting the possibility of an intertwined cardiomyopathic process rather than an isolated valvular affection. Furthermore, the lack of correlation with MAD and the presence of electromechanical dyssynchrony tend to point toward the left ventricular myocardium rather than the mitral valve. Nevertheless, a larger picture must be kept in mind integrating a wider spectrum of parameters, such as (1) clinical variables (PVC, lipothymia/syncope, and atypical chest pain), (2) valvular structural characterization (presence and severity of MAD and curling), (3) MVP-specific features (etiology, redundancy—single or bileaflet, and MR severity), and (4) myocardial substrate (strain analysis with postsystolic shortening and mechanical dispersion and pathognomonic late-gadolinium enhancement on CMR).

During follow-up, a majority of patients suffered from recurring arrhythmic episodes, mostly within the first year after the initial event, requiring appropriate ICD therapies. This observation points to the concept of a continual cardiomyopathic process and implies that ventricular arrhythmia in MVP has significant prognostic implications.

MVP must be carefully evaluated, particularly at the initial diagnosis, with a clinical focus on syncope or presyncope, and an evaluation of the extent of the prolapse, presence of MAD, and strain analysis must be carried out. PVC has to be regularly looked for using loop recorders. Further serial testing should be performed based on these initial results and in case of new symptoms and not solely on MR grade (19).

We believe that an analysis of myocardial deformation with strain echocardiography in MVP is valuable in routine practice and may help to identify patients in whom CMR may be pertinent. However, the association between postsystolic contraction, mechanical dyssynchrony, and myocardial fibrosis needs further evaluation.

### Study limitations

This study was limited by its retrospective design justified by the necessity of risk stratification and the rarity of the occurrence of SCD in MVP. The proportion of patients with SCD and MR grade repartition do not reflect the observed or expected proportion among the general population exposed to a potential risk of referral bias.

We acknowledge that strain measurements are prone to inter- and intraobserver variability (33). Postsystolic shortening may be witnessed in normal patients but with far lower values than in pathological myocardium. As for all indices derived from 2D acquisitions, care must be taken with the quality of images during acquisition and standardization in postanalysis to avoid false-positive PSI and incorrect MD measurement. The poorer reproducibility of these new indices is explained by the fact that they result from calculating at least two different parameters, thus increasing the margins of errors.

Cardiac MRI was systematically attempted in all aborted SCD patients but not always performed due to post-arrest related conditions. We also lack a complete evaluation among the otherwise normal MVP patients. At the present time, cardiac MRI is not routinely recommended for the assessment of MVP, and a specific study assessing the added value of LGE analysis on top of strain analysis in MVP should be undertaken.

## Conclusion

MVP remains an under-recognized cause of SCD, and the established risk factors appear insufficient to identify high-risk patients. Myocardial deformation and the presence of fibrosis are at the crossroads of myocardial–leaflet interactions and electrical ventricular hyperexcitability. Strain echocardiography, with identification of postsystolic shortening and increased mechanical dispersion values, conveys risk reclassification information of relative importance in the arrhythmic evaluation of MVP patients on top of established risk factors. Prognostic implications require further evaluation. Nevertheless, these parameters may be of particular interest in identifying high-risk MVP patients.

## Data Availability

The original contributions presented in the study are included in the article/**Supplementary Material**; further inquiries can be directed to the corresponding author/s.
