# Supplementary material for: Myocardial deformation in malignant mitral valve prolapse: A shifting paradigm to dynamic mitral valve–ventricular interactions
Source: Front Cardiovasc Med. 2023 Apr 12;10:1140216. doi: 10.3389/fcvm.2023.1140216 (PMC10130669; doi:10.3389/fcvm.2023.1140216)
Supplement: Supplementary file 1 [file Table1.docx]

Supplementary Material

**Table SM1:** Characteristics of patients with MVP and aborted SCD

| **Patient**  **n°** | **Sex** | **Age** | **Prior MVP Diagnosis** | **PVC** | **Pre / syncope** | **Ventricular arrhythmia** | **ST-T Changes** | **MR grade** | **Etiology** | **Bileaflet** | **MAD 0/1 (mm)** | **Curling 0/1 (mm)** | **GLS (%)** | **PSI** | **MD (ms)** | **LGE** | **ICD** | **Appropriate ICD Therapy** | |
| --- | --- | --- | --- | --- | --- | --- | --- | --- | --- | --- | --- | --- | --- | --- | --- | --- | --- | --- | --- |
| **1** | M | 46.1 | Yes | Yes | No | VT | Yes | 1 | FED | Yes | 0 | 0 | -18.2 | 5.46 | 56.3 | PM + M | Yes | 1 | 16 VT |
| **2** | F | 71.1 | Yes | No | No | VF | No | 2 | FED | No | 1 (9.1) | 1 (6.2) | -20.8 | 1.73 | 22.7 | M | Yes | 1 | 4 VT, 1 VF |
| **3** | M | 50.6 | No | Yes | No | VF | Yes | 4 | Barlow | No | 1 (9.2) | 1 (7.1) | -24.63 | 2.24 | 43.8 | - | Yes | 0 | - |
| **4** | F | 45.6 | No | Yes | Yes | VT | No | 2 | FED | Yes | 1 (12.3) | 1 (8.9) | -22.23 | 3.88 | 32.5 | No | No | - | - |
| **5** | M | 29.3 | Yes | No | Yes | VF | Yes | 1 | FED | Yes | 0 | 0 | -12.83 | 4.78 | 52.5 | PM + M | Yes | 1 | 1 VT |
| **6** | F | 52.3 | Yes | No | No | VT | No | 3 | FED | No | 0 | 0 | -17.8 | 7.93 | 45.5 | - | Yes | 1 | 10 VT, 1 VF |
| **7** | M | 15.1 | No | No | No | VF | Yes | 1 | FED | No | 0 | 0 | -21.73 | 1.46 | 25.7 | M | Yes | 0 | - |
| **8** | F | 35.1 | No | No | No | VF | Yes | 0 | FED | Yes | 0 | 0 | -17.73 | 4.97 | 61.3 | PM | Yes | 0 | - |
| **9** | M | 61.6 | Yes | No | No | VF | Paced | 2 | FED | No | 0 | 0 | -13.9 | 6.74 | 41.3 | - | Yes | 1 | 5 VF |
| **10** | M | 68.4 | No | Yes | No | VT | No | 2 | FED | No | 1 (1.1) | 1 (21.3) | -18.23 | 3.98 | 66.9 | PM + M | No | - | - |
| **11** | F | 60.6 | Yes | Yes | Yes | VT | No | 3 | Barlow | No | 0 | 0 | -22.33 | 5.17 | 42.8 | M | Yes | 1 | 174 VT, 8 VF |
| **12** | F | 68.5 | No | Yes | Yes | VF | No | 2 | FED | No | 0 | 0 | -14.8 | 3.67 | 39.2 | No | Yes | 1 | Numerous VT, VF |
| **13** | M | 47.8 | No | No | No | VT | No | 2 | Barlow | Yes | 1 (15.7) | 1 (9.9) | -18.37 | 6.77 | 70.0 | PM + M | Yes | 0 | - |
| **14** | F | 50.9 | Yes | Yes | No | VF | Yes | 1 | FED | Yes | 1 (10.6) | 1 (7.2) | -18.77 | 4.82 | 50.5 | - | Yes | 0 | - |
| **15** | F | 68.5 | No | No | No | VF | No | 1 | FED | No | 0 | 0 | -16.33 | 6.30 | 57.1 | PM + M | Yes | 0 | - |
| **16** | M | 21.1 | No | No | Yes | VF | No | 0 | FED | Yes | 0 | 0 | -15.63 | 4.65 | 40.4 | PM + M | Yes | 0 | - |
| **17** | F | 71.1 | No | No | No | VF | Yes | 3 | Barlow | Yes | 0 | 0 | -16.57 | 2.09 | 32.0 | PM + M | Yes | 1 | 32 VT, 1 VF |
| **18** | F | 23.3 | No | Yes | Yes | pVT | No | 2 | Barlow | Yes | 1 (10.5) | 1 (6.8) | -20.87 | 5.55 | 43.0 | M | No | - | - |
| **19** | M | 52.9 | No | Yes | No | VF | No | 4 | FED | Yes | 1 (8.8) | 1 (7.0) | -22.33 | 8.61 | 57.6 | - | Yes | 1 | 6 VT |
| **20** | F | 19.8 | Yes | Yes | No | VF | Yes | 1 | FED | Yes | 1 (11.9 | 1 (8.8) | -18.8 | 2.15 | 38.1 | No | Yes | 0 | - |

AP3C: Apical 3-chamber; ICD: Implantable cardioverter defibrillator; GLS: Global longitudinal strain; LGE: Late Gadolinium enhancement; MAD: Mitral annular disjunction; MD: Mechanical dispersion; MR: Mitral regurgitation; PLAx: Parasternal long axis; PSI: Post-systolic strain index; PVC: Premature ventricular contraction; pVT: Polymorphic ventricular tachycardia; SCD: Sudden cardiac death; VF: Ventricular fibrillation; VT: Ventricular tachycardia

**Table SM2:** Risk reclassification analysis based upon the classical risk factors for the risk of SCD.

|  |  | **NRI** | |
| --- | --- | --- | --- |
|  |  | **Index (CI 95%)** | **P values** |
| Model 1: classical risk factors (reference) | | |  |
| Model 2a | Model 1 + GLS | 31.5 (-14.1 to 77.1) | 0.18 |
| Model 2b | Model 1 + PSI | 60.7 (16.1 to 105.4) | 0.008 |
| Model 2d | Model 1 + MD | 70.6 (28.4 to 112.7) | 0.001 |
|  |  |  |  |
| Model 3 | Model 2d + PSI | 59.8 (15.2 to 104.5) | 0.009 |

Model 1: age, female sex, PVC, bileaflet prolapse, MR severity (> 2) and LVEF.

GLS continuous; MD in logarithmic scale; PSI dichotomous (PSI > 4).

GLS: Global longitudinal strain; MD: Mechanical dispersion; NRI: Net reclassification improvement; PSI: Post-systolic strain index.
